# C3orf21 ablation promotes the proliferation of lung adenocarcinoma, and its mutation at the rs2131877 locus may serve as a susceptibility marker

**DOI:** 10.18632/oncotarget.16798

**Published:** 2017-04-03

**Authors:** Litao Yang, Ying Wang, Meiyu Fang, Douhou Deng, Yongjun Zhang

**Affiliations:** ^1^ Department of Abdominal Surgery, Zhejiang Cancer Hospital, Hangzhou, China; ^2^ Department of Basic Medical Science, Zhejiang Chinese Medical University, Hangzhou, China; ^3^ Department of Integration of Traditional Chinese and Western Medicine, Zhejiang Cancer Hospital, Hangzhou, China

**Keywords:** C3orf21, cell apoptosis, cell migration, lung adenocarcinoma, polysaccharide modification

## Abstract

In this study, we investigated the role of C3orf21 gene polymorphism at the rs2131877 locus and its contribution to lung adenocarcinoma pathogenesis. Normal lung and tumor tissue sections were collected from fifteen patients with lung adenocarcinoma for chromosome 3 open reading frame 21 (*C3orf21*) genotype analysis. In addition, a retrospective analysis was performed to assess the association between C3orf21 genotype and tumor markers from patient samples used in our previously published study. In parallel, we also manipulated C3orf21 gene expression either by overexpressing or ablating it in a MSTO-211H human lung cancer cell line to further understand its contribution to cell proliferation, apoptosis and migration. Our results indicated that the patients with smoking history had a significantly increased mutation (rs2131877 T/C+C/C genotype) rate (*p* = 0.025), in addition to higher values for the CYF211 and NSE tumor markers (*p* = 0.014 and *p* = 0.031, respectively). The retrospective analysis also confirmed that the NSE marker value was higher in patients with a C3orf21 rs2131877 T/C+C/C genotype. Furthermore, our *in vitro* data indicated that C3orf21 ablation promoted lung cancer cell proliferation, inhibited apoptosis and accelerated cell migration. Overall, our study concluded that C30rf21 rs 2131877 T/C+C/C genotype patients may experience increased nicotine addiction and that C30rf21 can likely serve as a susceptibility marker for lung adenocarcinoma with a higher degree of malignancy.

## INTRODUCTION

Lung cancer has been the most common cause of cancer-related death worldwide [1] and appears to remain a major health problem in the near future. Non-small cell lung cancer (NSCLC) constitutes 82% of the total lung cancer cases [2] with a 5-year survival rate of approximately 15% in developed countries [3]. Further understanding of the NSCLC's molecular mechanism has led to the development of target therapies, which have improved overall survival, and the median OS has reached 3–5 years [4–11]. The Notch signaling pathway is one of many signaling pathways that have been shown to be important in NSCLC progression. The Notch protein has been shown to promote faster tissue growth and to up-regulate the self-renewal of the undifferentiated state of stem cells [12–20]. Notch activation has been shown to be stimulated by the differential O-linked glycosylation of its extracellular domain (NECD) [21], whereby O-linked glucose is added to a subset of epidermal growth factor-like (EGF) repeats in the NECD domain by protein O-glucosyltransferase 1 (POGLUT1) [22–24]. The chromosome 3 open reading frame 21 (*C3orf21)* gene encodes the Xyloside Xylosyltransferase 1 (XXYLT1) enzyme, which is involved in prolonging the O-linked xylose-glucose by activating the transfer of the second xylose into the notch extracellular protein EGF repetitive region and changing the Notch extracellular domain [25]. Our previous study showed that in the rs2131877 locus of the *C3orf21* gene, the frequency of the T/T homozygous genotype was higher in the peripheral blood of a healthy population than in adenocarcinoma patients (40.5% vs 29.7%) [26]. Thus, we undertook this study to determine if there is a link between the changes in the *C3orf21* gene mRNA expression and NSCLC development and its potential as a therapeutic target or a diagnostic molecular marker. We attempted to detect the mutation status of the rs2131877 locus in the C30rf21 gene by analyzing lung and adenocarcinoma tissues, respectively. We also attempted to identify the role of the C30rf21 gene in NSCLC using the high or low expressing lung cancer cell line MSTO-211H to detect its effects on cell proliferation, apoptosis and migration ability.

## RESULTS

### Correlation between patients’ clinical characteristics and C3orf21 rs2131877 genotype distribution

The 15 patients (7 males and 8 females) with lung adenocarcinoma were assessed for the genotype distribution of the C3orf21 rs2131877 locus. The analysis of 30 specimens (15 normal lung tissues and 15 tumor tissues) revealed that the frequencies of the rs2131877 T/C+C/C genotype were 20.0% and 33.3% in normal lung and adenocarcinoma tissues, respectively (Table 1).

**Table 1:** Clinical characteristics of patients

Patient	1	2	3	4	5	6	7	8	9	10	11	12	13	14	15
Sex	M	M	F	M	M	M	F	M	F	F	F	F	F	F	M
Age (years)	52	55	54	63	58	74	63	58	40	65	67	41	70	54	68
Smoking	yes	yes	No	yes	yes	yes	No	No	No	No	No	No	No	No	yes
Lung	TT	TT	TT	CC	TT	TC	CC	TT	TT	TT	TT	TT	TT	TT	TT
Cancer	TC	TC	CC	CC	TC	TT	TT	TT	TT	TT	TT	TT	TT	TT	TT

Abbreviations: M, male; F, female; Lung, lung tissue genotype; Cancer, cancer tissue genotype.

The clinical characterization indicated that the mean ages of the patients with the rs2131877 T/C+C/C (mutation) and rs2131877 T/T (wild type) genotypes were 56.40 and 60.00 years, respectively (Table 2). Further comparison between these genotypes indicated that 4 male and 1 female patient displayed the rs2131877 T/C+C/C genotype, while 3 male and 7 female patients had the rs2131877 T/T genotype; this difference was not statistically significant (*p* = 0.067) (Table 2). Additionally, the patients who smoke had a significantly increased rs2131877 T/C+C/C genotype compared to the rs2131877 T/T genotype (*p* = 0.025) (Table 2). Similarly, the values of CEA, CA125, CA199 and SCC-Ag tumor markers were also higher in tumors with the rs2131877 T/C+C/C genotype; however, the differences were not significant (Table 2). Notably, the values of the CYF211 and NSE tumor markers (4.18 ± 0.42 ng/ml and 25.46 ± 11.99 ng/ml, respectively) were significantly higher in the tumor patients with the rs2131877 T/C+C/C genotype (*p* = 0.014 and *p* = 0.031, respectively) (Table 2).

**Table 2:** Differences between *C3orf21* gene mutation and wildtype genotypes in tumor tissues

	Tumor Mutation patients (genotype: CC + CT)		Tumor Wildtype patients (genotype: TT)		*P* value
Ages	56.40 ± 4.28		60.00 ± 11.76		0.525
Gender	Male	Female	Male	Female	0.067
	4	1	3	7	
Smoking	Yes	No	Yes	No	0.025
	4	1	2	8	
CEA (ng/ml)	33.29 ± 60.65		8.91 ± 7.89		0.217
CA125 (U/ml)	59.06 ± 73.81		22.34 ± 20.50		0.155
CA199 (U/ml)	105.34 ± 204.17		24.79 ± 16.74		0.220
SCC-Ag (ng/ml)	1.64 ± 1.74		0.86 ± 0.45		0.192
CYF211 (ng/ml)	4.18 ± 0.42		1.81 ± 1.81		0.014
NSE (ng/ml)	25.46 ± 11.99		14.52 ± 5.86		0.031

We also performed a retrospective analysis of samples used in our previous study [26]. This analysis indicated that the mean age of the patients with the rs2131877 T/C+C/C (mutation) and rs2131877 T/T (wild type) genotypes were not significantly different (Tables 3, 4). Moreover, at the gender level, there were no significant differences between these genotypes (Tables 3, 4). Additionally, the CEA, CA125, CA199, SCC-Ag and CYF211 tumor markers did not show significant differences between patients with the rs2131877 T/C+C/C and rs2131877 T/T genotypes (Table 3). However, the values of the NSE tumor marker (15.46 ± 8.06) were significantly higher in the patients with the rs2131877 T/C+C/C genotype (*p* = 0.040) (Table 4).

**Table 3:** Differences between C3orf21 gene mutant and wildtype genotypes in patient's blood (*n* = 103)

	Patients with mutant genotype (genotype: CC + CT)		Patients with wildtype genotype (genotype: TT)		*P* value
Ages	57.01 ± 9.42		56.45 ± 6.90		0.765
Gender	Male	Female	Male	Female	0.699
	47	25	19	12	
CEA (ng/ml)	108.91 ± 374.01		101.17 ± 350.05		0.922
CA125 (U/ml)	197.41 ± 424.99		102.80 ± 303.15		0.265
CA199 (U/ml)	233.45 ± 716.61		440.13 ± 2149.34		0.467

**Table 4:** Differences between C3orf21 gene mutant and wildtype genotypes in patient's blood (*n* = 99)

	Patients with mutant genotype (genotype: CC + CT)		Patients with wildtype genotype (genotype: TT)		*P* value
Ages	57.16 ± 7.68		57.04±5.67		0.952
Gender	Male	Female	Male	Female	0.835
	45	24	17	10	
SCC-Ag (ng/ml)	1.69 ± 3.97		1.15 ± 0.91		0.488
CYF211 (ng/ml)	9.84 ± 16.35		11.26 ± 19.11		0.718
NSE (ng/ml)	15.46 ± 8.06		12.03 ± 4.43		0.040

### Assessment of C3orf21 gene manipulation effects on cell proliferation, apoptosis and migration of a human lung cancer cell line

**Figure 1:**
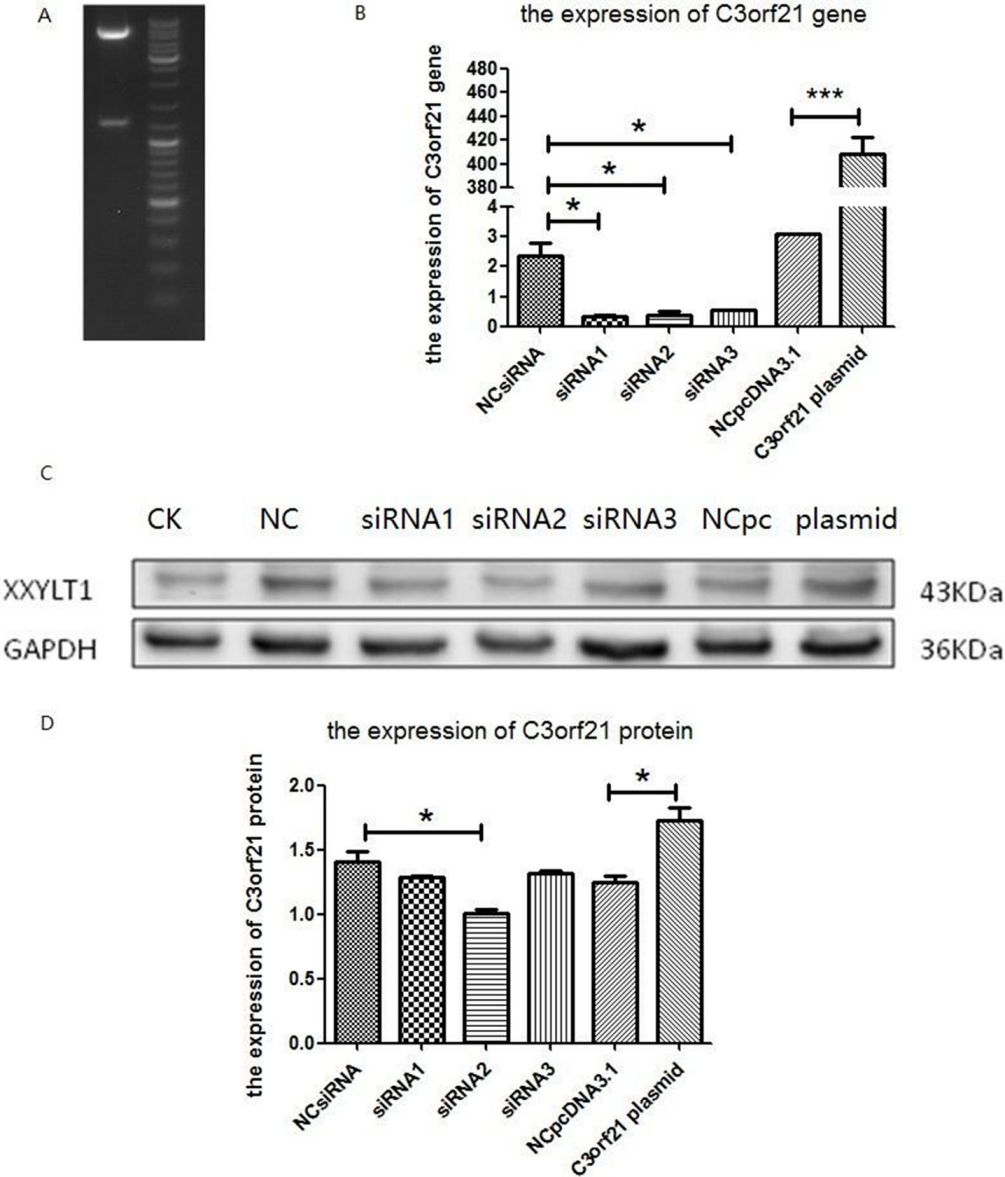
Confirmation of C3orf21 gene expression manipulation (Panel **A**) C3orf21 plasmid digested with HindIII and BamH1 restriction enzymes. (Panel **B**) the fluorescence quantitative PCR-based expression of the C3orf21 gene in MSTO-211H cells after its ablation with three siRNAs or overexpression. NC siRNA indicated that MSTO-211H cells were transfected with control siRNA; siRNA1, siRNA2 and siRNA3 indicated that MSTO-211H cells were transfected with three C3orf21 gene-specific siRNAs; NCpcDNA3.1 represented cells transfected with the control vector; and C3orf21 plasmid represented MSTO-211H cells overexpressing C3orf21 gene cDNA. * indicates a *P* value of < 0.05, while *** represents a *P* value of < 0.001. (Panel **C**) indicates the C3orf21 gene expression by western blotting. (Panel **D**) represents the normalized quantification of C3orf21 protein expression in different cell types. * indicates a *P value* of < 0.05.

To understand the effects of C3orf21 gene manipulation in NSCLC, we either ablated its expression with siRNA or overexpressed it in MSTO-211H, a human lung cancer cell line. To overexpress C3orf21, we successfully cloned C3orf21 plasmid, and its length was consistent with the expected size (Figure 1, panel 1A). All 3 siRNAs appear to significantly ablate the expression of the C3orf21 gene at the mRNA level, as determined using fluorescence quantitative PCR (Figure 1, panel 1B). Similarly, the overexpression of the C3orf21 gene using a pcDNA3.1-based vector in these cells also significantly elevated its expression compared to control vector after 48 hrs of transfection (Figure 1, panel 1B, final two bars). Additionally, the ablation and overexpression effects of the C3orf21 gene were also validated at the protein level using western blotting (panel 1C) and quantified after normalization with the GAPDH protein, as shown in panel 1D. Thus, overall, these results demonstrate that C3orf21 gene manipulations were efficient in MSTO-211H cells.

**Figure 2:**
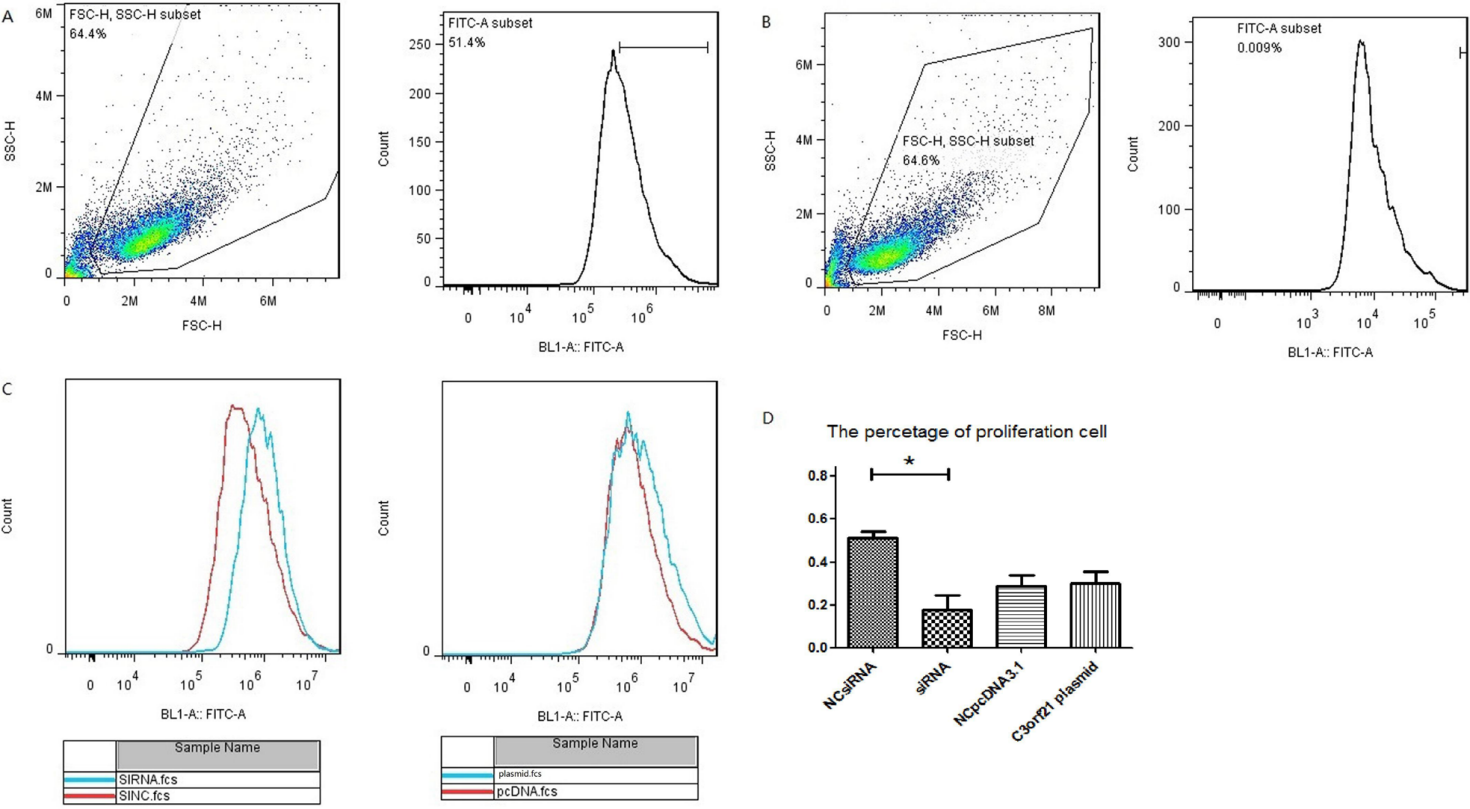
C3orf21 gene ablation promotes cell proliferation (Panel **A** and **B**) represent the strategy to assess cell proliferation by including MSTO-211H cell (A) and negative control (B). (Panel **C**) shows that C3orf21 gene ablation leads to faster MSTO-211H cell proliferation than normal cells, *p* < 0.05, while its overexpression has a very minimal effect. (Panel **D**) represents the quantification of the percent cell proliferation under different conditions. * indicates a *P* value of < 0.05.

Next, we assessed the effect of C3orf21 gene manipulations on MSTO-211H cell proliferation by measuring CFSE staining through flow cytometry. Our data revealed that siRNA ablation of the C3orf21 gene substantially promoted cell proliferation, while elevated C3orf21 gene expression did not show any significant reduction, as observed in Figure 2.

**Figure 3:**
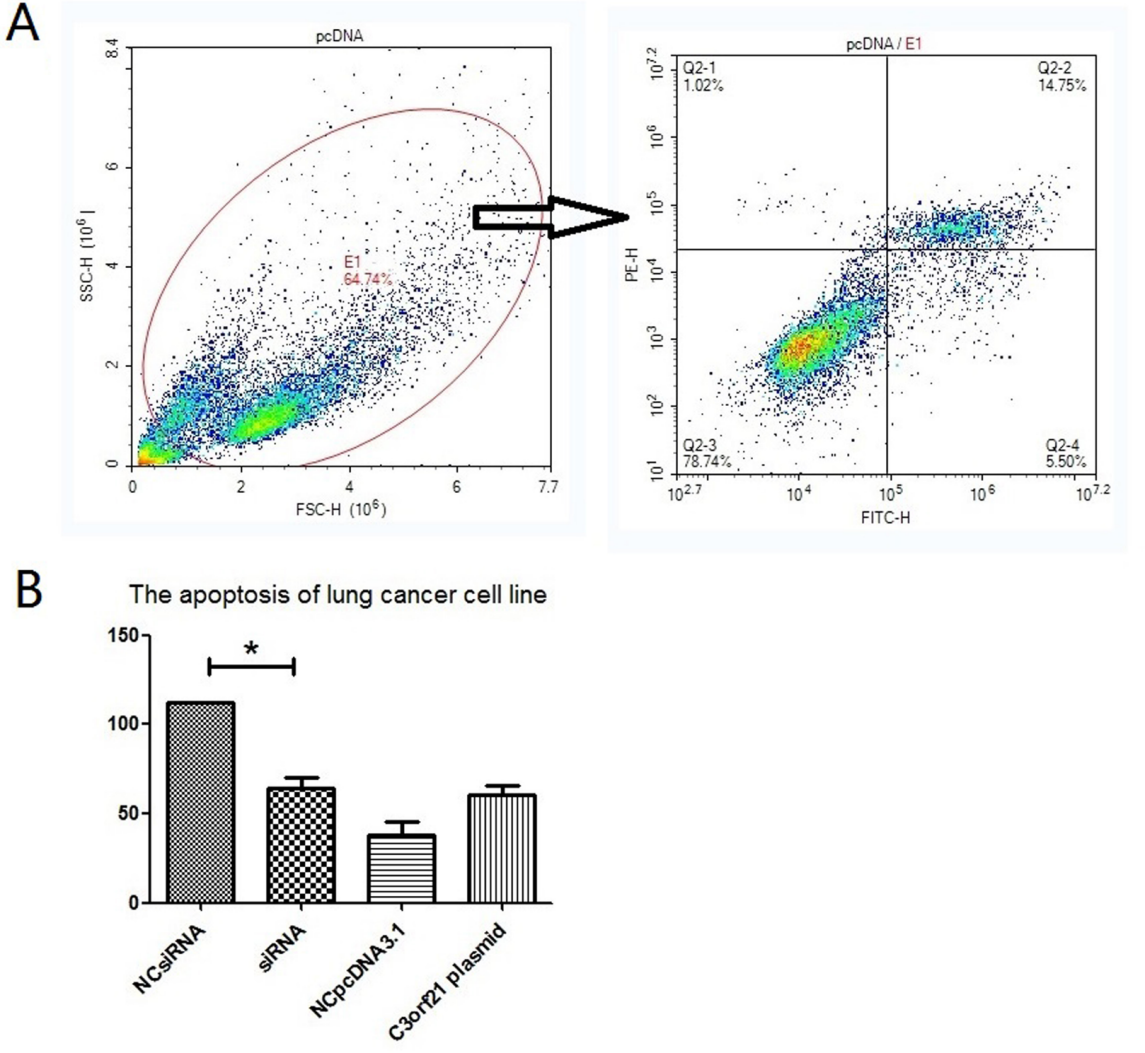
C3orf21 gene ablation inhibits cell apoptosis (Panel **A**) represents the strategy to assess percent cell apoptosis by flow cytometry. (Panel **B**) shows the comparison of percent cell apoptosis after C3orf21 manipulation. * indicates a *P* value of < 0.05.

We also analyzed the effect of C3orf21 gene manipulation on cell apoptosis using Annexin V and PI staining analysis via flow cytometry. Our results indicated that C3orf21 gene overexpression in MSTO-211H cells slightly accelerated the trend of lung cancer cell apoptosis; however, this accelerating effect was not significant compared to control vector (Figure 3, panel 3B). However, the siRNA-based ablation of C3orf21 gene expression significantly (*p* < 0.05) inhibited cell apoptosis observed in Figure 3 (panel 3B, first two bars).

**Figure 4:**
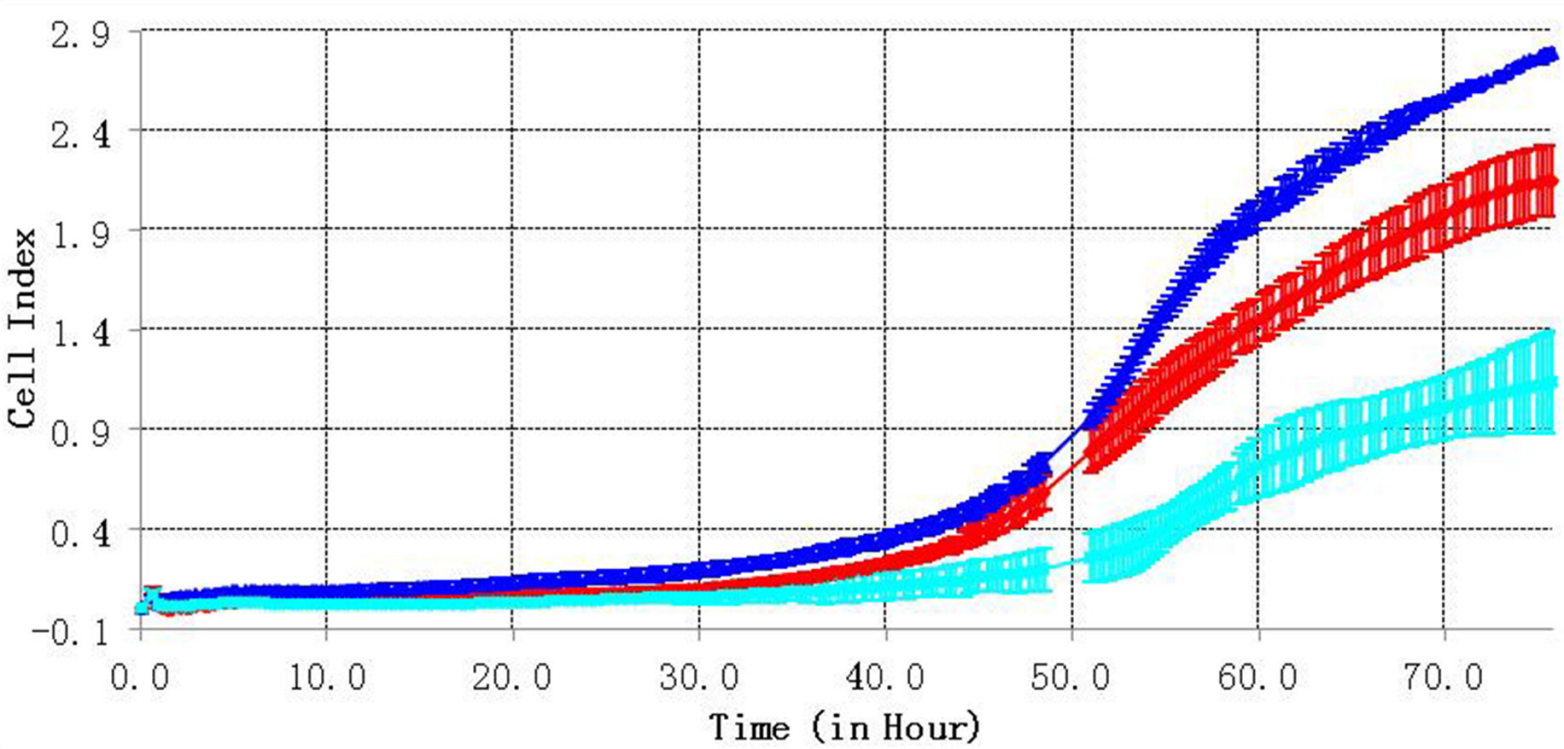
C3orf21 gene ablation accelerates cell migration The red curve represents the migration of the MSTO-211H control group cells, the blue line represents cells from the C3orf21 ablation group, and the light blue line indicates cells from the C3orf21 overexpression group.

We also tested the effect of C3orf21 gene manipulation on the cell migration ability of MSTO-211H cells using RTCA detection technology, which measured cell migration every 15 minutes over a period of 72 hrs. Our results indicated that C3orf21 ablation enhanced cell migration (blue curve), while its overexpression inhibited migration (light blue curve) compared to control cells (red curve), as observed in Figure 4.

## DISCUSSION

Based on our analysis, lung adenocarcinoma tissues displayed a lower frequency of the T/T homozygous genotype (wild-type) in the C3orf21 gene rs 2131877 locus compared with normal lung tissue (66.7% vs 80%). Additionally, the values for tumor markers, including CYF211 and NSE, were higher in patients with the rs 21319877 T/C+C/C genotype in tumor tissue, and NSE was higher in patients with the rs 2131877 T/C+C/C genotype in blood. These observations indicate specific differences between the clinical characteristics of lung adenocarcinoma patients with different genotypes. The high expression levels of the NOTCH1 and NOTCH3 genes have been observed to be significantly associated with poor prognosis in lung adenocarcinoma [27, 28]. Additionally, DLL4 (Delta-like 1, one of five Notch ligands) was observed to be positively associated with poor OS in NSCLC patients [29]. More specifically, patients exhibiting high Notch 1, Notch 3, JAG1 (Jagged, one of five Notch ligands) or DLL4 protein levels had a significantly worse OS rate [30]. In this context, our results also suggested that the C30rf21 rs 2131877 T/C+C/C genotype may act as a marker for higher malignant adenocarcinoma. Moreover, we also observed that patients who smoke had a significantly increased rate of the C30rf21 rs 2131877 T/C+C/C genotype. However, smoking was not substantially associated with adenocarcinoma; this indicated that the C30rf21 rs 2131877 T/C+C/C genotype patients may have higher nicotine addiction. The number of patients was small in our study; thus, the results were difficult to conclude decisively. A large study is required to validate our observation.

Furthermore, the Notch signaling pathway has been demonstrated to up-regulate cell proliferation and control differentiation and apoptotic processes [31]. The reduced activity of UDP-xylose:-xyloside 1,3-xylosyltransferase (XXYLT1), which is encoded by the C3orf21 gene [29], would lead to enhanced Notch signaling [32]. Notch signaling participates in TIC (tumor-initiating cells) fate in NSCLC [33, 34], and Notch deregulation has been reported to be associated with the development of lung cancer [35]. However, Notch signaling inhibitors exhibited potent antitumor activity in the lungs [36]. Our study revealed that the ablation of the C3orf21 gene (XXYLT1) promoted lung cancer MSTO-211H cell proliferation, inhibited apoptosis and accelerated cell migration. However, its overexpression did not significantly alter cell proliferation or apoptosis, and this could be attributed to the fact that C3orf21 (XXYLT1) over-expression in Notch signaling was dependent on the specific tissue context, microenvironment, and crosstalk with other signaling pathways. The Notch 1 protein has also been known to suppress tumor proliferation under normoxia in lung cancer; however, it exhibited the opposing role of tumor promotion under hypoxia conditions [37]. Additional research is required to elucidate this aspect.

## MATERIALS AND METHODS

### Patient selection and C3orf21 rs2131877 genotype identification

Fifteen patients (seven male and eight female) who were newly diagnosed with lung adenocarcinoma and underwent surgical treatment between March 2008 and November 2009 at Zhejiang Cancer hospital were recruited for this study. All subjects provided informed consent, and the study was approved by the ethics committee of Zhejiang Cancer Hospital. A total of 15 normal lung tissues and 15 cancer tissues were obtained from these patients during surgery and were stored at −80°C for further analysis. The droplet Digital PCR (dd-PCR) method was used to evaluate the genotype of C3orf21 in normal lung tissue and adenocarcinoma specimens. The DNA was isolated from these lung and tumor tissue homogenates, respectively, using the FastPrep-24 sample processing system (MP Biomedical, Santa Ana, California, USA), and the genotyping was performed with the PCR sequencing method using an S100-PCR Amplification Device (Bio-Rad, Samoa, USA). The assay designer's software version 3.0 (Sequenom) was used to design primers for the polymerase chain reaction and single base extension. The primers for the C3orf21 rs2131877 genotype were:

1st, 3′-CTGGCCTTCAGGACCTAC-5′

2nd, 3′-CTGCCTGTAACCTCAAAGA5′

Finally, the purified PCR products were sequenced using an ABI 3100 DNA sequencer (USA). Other tumor markers, such as carcinoembryonic antigen (CEA), carbohydrate antigen 125 (CA125), cell keratin fragments 211 (CYF211), carbohydrate antigen 199 (CA199), squamous cell antigen (SCC-Ag), and neuron enolization enzymes (NSE), were detected in the peripheral blood before surgery with a Chemiluminescent Microparticle Immunoassay in the Abbott I400 AxSYM system (Abbott, Chicago, USA). All patients were followed up until June 2016. We also performed a retrospective analysis of two hundred NSCLC patients including 145 patients with adenocarcinoma and 55 patients with squamous cell carcinoma from our previous study [26]. All of these patients were non-smokers. Among these, one hundred and three adenocarcinoma patients displayed tumor markers (CEA, CA125, CA199) (Table 3); however, only ninety-nine patients had records for the values of all markers (CEA, CA125, CA199, SCC-Ag, CYF211 and NSE) (Table 4).

### Cell culture and C3orf21 overexpression and ablation

The MSTO-211H human lung cancer cell line, which was procured from the cell bank of the Chinese Academy of Sciences Library/Stem Cells, was cultured in a humidified incubator with 5% CO_2_ at 37°C in six-well plates (1 × 10^6^ cells/ml) containing RPMI-1640 media (Gibco, Gaithersburg, MD, USA) supplemented with 10% fetal bovine serum (Gibco, Gaithersburg, MD, USA). To perform C3orf21 gene ablation, the following three siRNAs sequences were designed by the bioinformatics software Primer Premier 5.0 (Premier Biosoft International, Palo Alto CA, USA):

siRNA1 sense: 5′-GGAACUGCAACACUCCCAU TT-3′ and antisense: 5′-AUGGGAGUGUUGCA GUUCCTT-3′; siRNA2 sense: 5′-GCAUCAGAU CAUGCCCAAATT-3′ and antisense: 5′-UUUGGGCAUG AUCUGAUGCTT-3′; siRNA3 sense: 5′-GCACGAGGUG CUUAACCUUTT-3′ and antisense: 5′-AAGGUUAAG CACCUCGUGCTT-3′; Similarly, to overexpress the C3orf21 (XXYLT1) gene, it was cloned into pcDNA3.1 plasmid using HindIII/BamHI sites with T4 DNA ligase (Figure 1, panel A). The ligation product was further transformed into a competent Escherichia coli strain, and positive clones were selected by ampicillin selection and then sequenced using ABI3730 sequencing analysis (Sangon, Shanghai, China). The siRNAs were transfected into an MSTO-211H cell line with RNAiMAX (Lipofectamine) transfection agent, while the plasmid overexpressing C3orf21 cDNA was transfected using Lipo3000 reagent. The C3orf21 gene ablation or overexpression was verified at the mRNA and protein levels using quantitative PCR and western blotting, respectively.

### RNA isolation, reverse transcription, and PCR

The RNA was isolated from MSTO-211H cells either ablated for or overexpressing the C3orf21 gene using an RNAiso Plus (TaKaRa, Dalian, China) kit. To generate cDNA, the isolated RNA (1 μg) was reverse transcribed using an RT transcription kit (TaKaRa, Dalian, China) with oligo (dT18) primer. Next, the quantitative PCR was conducted using a real time qPCR (Bio-Rad, USA) machine with the following specific primers synthesized by Sangon Biotech (Shanghai, China): 5′-CTGGCCTTCAGGACCTAC-3′ and 5′-CTGCCTGTAACCTCAAAGA-3′. The quantitative PCR reaction mix consisted of 20 μl total volumes including 10 μl SYBR^®^ Premix Ex Taq (TaRaKa), 0.8 μl of each primer (10 μM), 0.2 μl cDNA, and 6.4 μl of ddH2O. The following PCR amplification conditions were used: denaturation at 95°C for 30 seconds, then 40 cycles of 95°C for 5 seconds and 60°C for 20 seconds, and a final cycle of 72°C for 1 minute. For all PCR analyses experiments, the GAPDH expression served as a loading control.

### Western blotting

Cells were collected and lysed on ice for 30 min in RIPA lysis buffer (cell-land, Hangzhou, China). Protein fractions were collected by centrifugation at 15,000 g at 4°C for 10 min, were subjected to 10% SDS-PAGE and transferred to PVDF membranes (Bio-Rad, USA). After blocking with 5% BSA, the membranes were incubated with specific antibodies overnight at 4°C. The following day, a secondary antibody was added, and the proteins were visualized using an enhanced chemiluminescence detection system as recommended by the manufacturer Bio-Rad Laboratories, Inc.

### Cell proliferation analysis

The 1 × 10^6^ cells (MSTO-211H) per ml in a logarithmic growth phase were incubated with 10 μm CFSE staining solution at 37°C for 30 min. These cells were later washed twice with PBS and then cultured in a humidified incubator with 5% CO_2_ at 37°C in six-well plates. After 72 hr, the cells were collected and analyzed for cell proliferation using flow cytometry detection technology.

### Apoptosis analysis

Following incubation for 24 hrs, MSTO-211H cells were incubated with 5 μl of FITC-conjugated Annexin V dye and 10 μl of PI staining reagent in the dark for 5 min at room temperature. These cells were then immediately analyzed using flow cytometry.

### Assessing cell migration using real time cell analysis (RTCA)

To assess the cell migration of MSTO-211H cells, we performed a real time cell analysis. First, 165 μl serum containing culture medium was added to the lower chamber of the cell invasion and migration (CIM) plate, while 30 ul of serum-free medium was added to the upper chamber, and the plate was incubated at 37°C and 5% CO2 for 1 hr to balance the baseline. Next, 100 μl of the logarithmic phase cells (concentration 4 × 10^5^ per ml) in serum-free medium were added into the CIM upper chamber, and the cell migration was detected every 15 min. The real-time cell migration data were collected for 72 hrs.

### Statistical analysis

The flow cytometry data were analyzed using FlowJo 7.6 software. The results were analyzed using the statistical software GraphPad Prism 5.0 and presented as the means ± standard error for continuous variables. An analysis of variance and the following independent *t* tests were used for all comparisons. Correlations between two variables were estimated using Spearman's rank sum test, and a *P* value of < 0.05 was considered statistically significant.

## CONCLUSIONS

In summary, we demonstrated that the C3orf21 gene T/C+C/C genotype at the rs 2131877 locus may act as a susceptibility marker for lung adenocarcinomas with a higher degree of malignance. Importantly, C3orf21 mRNA expression appears to be associated with lung cancer risk, and its ablation promoted lung cancer MSTO-211H cell proliferation, inhibited apoptosis and accelerated cell migration.
